# Intrahepatic levels of microbiome-derived hippurate associates with improved metabolic dysfunction-associated steatotic liver disease

**DOI:** 10.1016/j.molmet.2024.102090

**Published:** 2024-12-31

**Authors:** Maxime Deslande, Francesc Puig-Castellvi, Inés Castro-Dionicio, Romina Pacheco-Tapia, Violeta Raverdy, Robert Caiazzo, Guillaume Lassailly, Audrey Leloire, Petros Andrikopoulos, Yasmina Kahoul, Nawel Zaïbi, Bénédicte Toussaint, Frédérik Oger, Nicolas Gambardella, Philippe Lefebvre, Mehdi Derhourhi, Souhila Amanzougarene, Bart Staels, François Pattou, Philippe Froguel, Amélie Bonnefond, Marc-Emmanuel Dumas

**Affiliations:** 1 University of Lille, Lille University hospital, 59045, Lille, France; 2 INSERM U1283, CNRS UMR 8199, Institut Pasteur de Lille, 59045, Lille, France; 3 Division of Systems Medicine, Department of Metabolism, Digestion and Reproduction, Imperial College London, London, W12 0NN, United Kingdom; 4 INSERM U1190, Institut Pasteur de Lille, University of Lille, Lille University Hospital, 59045, Lille, France; 5 INSERM U1011 Institut Pasteur de Lille, University of Lille, Lille University Hospital, 59045, Lille, France; 6 The Victor Phillip Dahdaleh Institute of Genomic Medicine, McGill University, Montréal, H3A 0G1, Canada

**Keywords:** MASLD, Metabolic diseases, Metabolome, Microbiome, Hepatocyte, RNAseq

## Abstract

**Objective:**

Metabolic dysfunction-associated steatotic liver disease (MASLD) is characterised by lipid accumulation in the liver and is often associated with obesity and type 2 diabetes. The gut microbiome recently emerged as a significant player in liver metabolism and health. Hippurate, a host-microbial co-metabolite has been associated with human gut microbial gene richness and with metabolic health. However, its role on liver metabolism and homeostasis is poorly understood.

**Methods:**

We characterised liver biospies from 318 patients with obesity using RNAseq and metabolomics in liver and plasma to derive associations among hepatic hippurate, hepatic gene expression and MASLD and phenotypes. To test a potential beneficial role for hippurate in hepatic insulin resistance, we profile the metabolome of (IHH) using ultra-high-performance liquid chromatography coupled to high-resolution tandem mass spectrometry (UHPLC-MS/MS), and characterised intracellular triglyceride accumulation and glucose internalisation after a 24 h insulin exposure.

**Results:**

We first report significant associations among MASLD traits, plasma and hepatic hippurate. Further analysis of the hepatic transcriptome shows that liver and plasma hippurate are inversely associated with MASLD, implicating lipid metabolism and regulation of inflammatory responses pathways. Hippurate treatment inhibits lipid accumulation and rescues insulin resistance induced by 24-hour chronic insulin in IHH. Hippurate also improves hepatocyte metabolic profiles by increasing the abundance of metabolites involved in energy homeostasis that are depleted by chronic insulin treatment while decreasing those involved in inflammation.

**Conclusions:**

Altogether, our results further highlight hippurate as a mechanistic marker of metabolic health, by its ability to improve metabolic homeostasis as a postbiotic candidate.

## Highlights

• Circulating and hepatic hippurate anticorrelate with genes involved in MASLD.
• Hippurate correlates with metabolites involved in energy homeostasis and inflammation regulation in hepatocytes.
• Hippurate modulates the hepatocyte metabolome and inhibits hepatic steatosis.
• Hippurate rescues insulin resistance induced by chronic insulin treatment.

## 1 Introduction

More than 1 billion people (or 1 in 8) live with obesity, and diabetes currently affects 650 million adults (1.3 billion estimated by 2050) [1,2]. This epidemic also fuels the rise in metabolic dysfunction-associated steatotic liver disease (MASLD, previously known as NAFLD [3,4]). Although often considered benign, intra-hepatic lipid accumulation is an independent risk factor for type 2 diabetes and cardiovascular diseases [5]. A severe complication of obesity is metabolic dysfunction-associated steatohepatitis (MASH, previously known as non-alcoholic steatohepatitis, NASH) characterised by liver inflammation, fibrosis (brunt) [6] and abnormal cell swelling (ballooning) [7]. MASLD and MASH are assessed by the NAFLD activity score (NAS) [8]. Remarkably, 30–40% of obese subjects resist the development of liver steatosis [9], and the reason behind this MASLD protection remain elusive.

The gut microbiota and its collective genome (i.e., the microbiome) play a significant role in cardiometabolic diseases [[10], [11], [12], [13], [14]], such as obesity [15], diabetes [16], and MASLD [17]. This microbiome has multiple mutualistic interactions with its host, including through the production of biologically active metabolites such as short-chain fatty acids, bile acids, or aromatic amino acid metabolites (such as imidazoles, indoles, and cresols) [18,19]. Compounds such as short chain fatty acids that are derived from microbial metabolism and beneficial for the host are now known as postbiotics [20,21]. In particular, the phenylpropanoid pathway has gained interest over the last two decades [22], and its end-product hippurate, the ionic solute of hippuric acid at physiological pHs, has been repeatedly reported as a host-beneficial metabolite for metabolic health [[23], [24], [25]]. Hippurate is the product of co-metabolism between the microbiota and their host involving phase 2 conjugation of microbial benzoate synthesized in the phenylpropanoid pathway with glycine mainly in hepatocytes (and also to a lower extend in the kidney mitochondrial matrix [26,27]), before being released in systemic circulation and excreted by the kidneys [28,29].

Hippurate is considered a postbiotic candidate: we and others have shown that hippurate has been positively associated with the gut microbial gene richness, alpha diversity, associates with metabolic health in adults and improves glucose tolerance in mice on a high-fat diet (HFD) while stimulating insulin production and β-cell proliferation [30]. Circulating levels of hippurate are also negatively correlated to hepatic steatosis [25] and metabolic syndrome [23]. This suggests that hippurate contributes to maintaining metabolic homeostasis. However, although published literature points towards an active role on hepatic metabolism, it is unclear whether hippurate directly modulates liver homeostasis and hepatocyte metabolism.

Here, we show in patients with obesity having a range of MASLD phenotypes that both circulating and intrahepatic hippurate negatively associates with steatosis, inflammation, and characteristic MASLD and MASH gene expression patterns, while they associate positively with glucose tolerance. We show that hippurate is internalized in hepatocytes, paving the way for its mechanistic *in vitro* study. Furthermore, we show that hippurate rescues insulin-induced lipid accumulation in a human hepatocyte cell line while inhibiting glucose uptake.

## 2 Materials and methods

### 2.1 PreciNASH [31]

The cohort Biological Atlas of Severe Obesity (ABOS) has been characterised in-depths with a collection of metabolic tissues (liver, muscle, visceral and subcutaneous adipose tissues, intestine) and blood samples (plasma, serum, DNA) collected as part of an ongoing clinical trial (NCT01129297). The PreciNASH (PreciNASH ANR-16-RHUS-006) project, aims to develop predictive models of MASH in order to predict disease progression in at-risk patients and evaluate the long-term effects of targeted treatments for precision medicine [31]. For the present study, we studied a subset of 318 patients whose hippurate levels were measured in both liver and serum. Patients with a steatosis score of ≥1 (indicating >5% of liver surface area affected by steatosis) were classified as MAFLD patients. Among these, patients presenting both hepatocyte ballooning and inflammation were classified as having MASH [30]. According to this classification, 146 patients suffered from non-MASH MASLD and 81 patients had MASH (Table S1).

#### 2.1.1 RNA-seq

RNA was extracted from liver biopsies and was performed using the KAPA RiboErase Kit (Human, mouse, Rat; Roche Sequencing) and KAPA RNA HyperPrep Kit (Roche Sequencing). We extracted a total of 380 RNA samples, starting with 1.8 μg of RNA per sample. RNA quality was assessed using the RNA Integrity Number (RIN), with values ranging from 8.5 to 2.25. Of these samples, 96 had a RIN >7, 192 had a RIN between 5 and 7, and 92 had a RIN <5. Despite the variability in RNA quality, total RNA-seq was performed to accommodate samples with lower RIN values. The libraries were sequenced in 2 × 75 bp paired-end reads with the NovaSeq6000 Illumina system. On average, more than 72 million reads per sample were generated, and more than 95 % were accurately mapped to the human genome (hg38). Demultiplexing of sequence data was performed using bcl2fastq Conversion Software (Illumina; version v2.20.0.422). The QC was performed using the FastQC software (version v0.11.9). The removal of adaptor sequences and low-quality bases was performed with Trimmomatic (version v0.39). Sequence reads were then mapped to the human genome (GRCh38) using Star aligner (version v2.7.3a). The raw and normalised counting steps were computed using RSEM v1.3.0 with a GTF file from GENCODE version 38. Ensembl release 104 was used to assign gene IDs for the RSEM counts. These counts were then read using the tximport R package and normalised using method VST from the R package DESeq2. For every patient, the Transcripts Per Million (TPM) data of transcripts from 60,649 different genes was obtained. TPM values below 1 were excluded from further analysis in all patients.

#### 2.1.2 Untargeted metabolomics

Plasma and liver samples were prepared and profiled by Metabolon (Durham, NC) using UHPLC-MS/MS described in detail in 2017 by Long T. et al. in Nature Genetics [32]. Hepatic samples were extracted as described in detail in 2023 by Raverdy V. et al. in Obesity [31].

Annotated metabolites and unknown features were identified by comparing sample features with a ion features in a reference database of pure chemical standards and previously detected unknowns, followed by detailed visual inspection and quality control as in 2010 by Dehaven C.D. et al. in Journal of Cheminformatics [33]. It was performed by Metabolon, Inc. (Morrisville, USA).

### 2.2 Cell culture and treatments

Human Immortalized Hepatocytes (IHH), supplied by the UMR1283/8199 [34], were expended and maintained as monolayer in William’s E medium in William’s E medium (Thermo Fisher Scientific, 12551032) supplemented with 10% FBS, 1% MEM Non-Essential Amino Acids Solution (Thermo Fisher Scientific, 11140035), 10 mM HEPES (Thermo Fisher Scientific, 15630056), 50 μM 2-mercaptoethanol (Gibco™ 31350010) and 50 U/mL penicillin/streptomycin (Thermo Fisher Scientific, 15140122) at 37 °C under 5% CO2. Cells were used between passages 29 and 35. Cells were passed 2 times per week by ¼. When needed, cells were treated either with hippuric acid at 500 μM resuspended in DMSO. Cells were also treated with insulin (Sigma–Aldrich, I9278) at 100 nM in chronic (24 h) and 200 nM in acute (1 h) experiments.

### 2.3 Triglyceride dosage

Lipids droplets were quantified using the Oil Red O. After 24 h of hippurate treatment (DMSO for control) and/or with 100 nM insulin (water for control), IHH cells were washed with PBS at room temperature prior to a fixation with 4% formaldehyde during 30 min. Fixed cells were washed with PBS and then permeabilised for 5 min using 60% isopropanol. The isopropanol was removed and permeabilised cells were incubated in a filtered 60% Oil Red O solution for 15 min. The cells were washed 5 times with PBS before being placed back in PBS to take photos (Invitrogen™ EVOS™ XL Core Imaging System, 15339661). The cells were lysed for 5 min in gentle agitation at room temperature in 100% isopropanol. Following Oil Red O extraction, the absorbance of 500 μL of the suspension, was read at 490 nm with Glomax Discoverer from Promega (GM3000). The absorbance of each well was then normalised to the number of cells (thanks to photos) in the respective wells by the Image J (RRID:SCR_003070) software.

### 2.4 Glucose uptake assay

Glucose uptake was estimated using the Glucose colorimetric Detection Kit from Invitrogen™ (EIAGLUC) following the manufacturer’s instructions. Culture medium was collected at 3 time points: T (0) medium in contact with the cells, T (24) after a cell treatment with hippurate (DMSO for control) and/or 100 nM insulin (water for control) during 24 h and T (25) after 1 h subsequent treatment with 200 nM insulin (in addition of hippurate and/or insulin already used). These culture medium samples were diluted 1:15 with the kit’s assay buffer to ensure that the glucose concentration of the samples falls within the calibration curve. These diluted samples were placed in a 96-well plate. Horseradish Peroxidase Concentrate (HRP) at 1X, Substrate and Glucose Oxidase at 1X were then added to each sample. Samples were incubated for 30 min at room temperature. Then, the absorbance was read at 560 nm with Glomax Discoverer (Promega, GM3000).

### 2.5 Metabolomics

#### 2.5.1 Sample preparation

IHH cells were treated with hippuric acid (DMSO for control) either for 1 h (for targeted metabolomics to test hippurate entering cells) or for 24 h (for untargeted metabolomics to investigate the effects of hippurate on hepatocyte metabolism). After treatment, the medium was removed and the cells were washed once with complete medium. The cells were placed in contact with 100% methanol containing 40 ng/mL hippuric acid-d5 (HA-D5) at −20 °C and then resuspended by scraping in order to be transferred to 1.5 mL microtube. The cells were vortexed twice for 15 s and then centrifuged at 14,000 g at 4 °C for 5 min. The sample supernatant containing metabolites extracted from the cell was transferred to a new microtube before being evaporated for 3 h at 37 °C [[35], [36], [37]]. Dry samples were stored at −20 °C overnight and subsequently reconstituted with 5% v/v MeOH in water.

#### 2.5.2 UHPLC-HRMS

An external calibration is performed on the spectrometer before each analysis with Pierce™ FlexMix™ Calibration solution (Thermo Fisher Scientific, Bremen, DE). Chromatographic analysis was performed using a Vanquish DUO UHPLC system (Thermo Fisher Scientific, Bremen, DE). Sample were injected into Thermo Scientific™ Hypersil GOLD™ column (15 cm × 2.1 mm ID, 1.9 μm particle size) at 40 °C. Mobile phases consisted of (A) H_2_O with 0.1% formic acid and (B) methanol with 0.1% formic acid. The injection volume was 5 μL. After separation by UHPLC, mass spectrometry was performed using the Exploris 240 (Q-Orbitrap, Thermo Fisher Scientific, Bremen, DE) with an electrospray ionization (ESI) source (Thermo Fisher Scientific, Bremen, DE) operated in positive and negative ionization modes. Static spray voltage was set at 3500 V for positive ion and 3000 V for negative ion, static gas mode, sheath gas at 40, aux gas at 8, sweep gas at 1, ion transfer tube temperature at 275 °C, vaporizer temperature at 320 °C. Full scan properties were as follows: orbitrap resolution at 60,000, RF lens at 70%, data type profile/centroid and intensity threshold at 10^5^. For both ionisation modes, acquisition was performed from *m*/*z* 67 to 1000. An internal mass calibration is performed before each injection with Fluoranthene (Thermo Fisher Scientific, Bremen, DE). MS/MS spectra were obtained in data dependent mode (DDA) with 5 dependent scans. The MS/MS parameters were: isolation windows *m*/*z* 2, normalised collision energy type, HCD collision energies 30 V, 50 V and 150 V, orbitrap resolution at 30,000, scan range mode automatic and centroid data type [38]. The pooled samples were analysed using the DeepScan LC/MS strategy. The experimental parameters used were an exclusion factor of 10, an exclusion and inclusion list peak window extension of 1 s, an inclusion list peak fragmentation threshold of 50% and an exclusion time of 10 s. In positive mode, the preferred ion was [M+H]^+^, while in negative mode, the preferred ion was [M−H]^-^. Each pooled sample was analysed in the following sequence: 2 header blanks and 6 sample injections. The two blanks and the first sample injection were measured in full scan mode, while the other samples were measured in DDA mode. The second blank was used to create the first exclusion list, while the first sample injection was used to create the first inclusion list.

#### 2.5.3 Data pre-processing

UHPLC/HRMS data were processed separately for each LC/MS method using MS-DIAL 4.9 software [39]. The DeepScan of each pooled sample and each LC/MS method was also processed separately with MS-DIAL. MS1 and MS2 tolerances were set at 0.0015 and 0.05 Da, respectively. Peaks were aligned to a QC reference file with a retention time tolerance of 0.1 min and a mass tolerance of 0.0015 Da. Feature annotation (*m*/*z* - retention time pairs) was performed using the GNPS spectral database (public spectral database (MSI level 2)) and IROA (internal spectral database (MSI level 1)) with MS1 and MS2 tolerances of 0.01 and 0.05 Da, respectively. The peak area tables resulting from the MS-DIAL pre-processing of the LC/MS data described above were refined as follows: 1) MS2 data and annotations from samples analysed using the DeepScan approach were transferred to LC/MS features without MS2 (using as matching criteria a mass tolerance of 5 ppm and an average retention time between the left and right limits of the features detected in the DeepScan runs). 2) International Chemical Identifier (InChIKeys), molecular formula was added for annotated peaks. 3) MS-DIAL adducts were corrected according to these formulae. 4) For each annotated compound, only the adduct with the largest average peak area was retained. 5) Next, the MS-DIAL project data (positive and negative) were combined. 6) Finally, the IUPAC name and chemical classes (ClassyFire) were added. Prior to statistical analysis of the data, the peak area table was filtered to include only the areas of features with less than 30% RSD in the QC samples. Finally, the data were normalised by probabilistic quotient (PQN) and auto scaling. This was carried out using R Statistical Software (v4.1.2; R Core Team 2021). This resulted in 12,096 features, including both annotated and non-annotated, with 250 unique metabolites identified.

#### 2.5.4 Targeted isotopic quantification

All chemical standards, including the Authentic Analytical Standard (ASD) and the Internal Standard (ISD), were in granular powder form on delivery to the laboratory and were stored at −20 °C until used. Stock solutions of individual standards were prepared by weighing the original powder to an accuracy of 0.1 mg on an OHAUS Semi Micro analytical balance (OHAUS Pioneer, OHAUS Europe GmbH, Nänikon, Switzerland) and dissolving in MeOH to a concentration level of 100 μg/mL. The ∗.RAW data acquired in full scan mode were manually inspected in XCalibur Qual browser version 4.3 (Thermo Fischer Scientific). Automatic batch peak integration was performed using Tracefinder 5.1 software (Thermo Fischer Scientific, Waltham, MA, USA). Standard calibration solutions from 0.37 μM to 11.7 μM were prepared, and a calibration curve with 6 consecutive points was generated for HA, with the ratio of the peak area of the ASD ion (HA) to the ISD (HA-D5) on the y-axis and the ratio of the ASD quantity to the ISD on the x-axis. The peak area self-integration results in TraceFinder were manually checked in XCalibur.

### 2.6 Statistical analysis

#### 2.6.1 Human data

Age and sex were used as covariates in all PreciNASH analyses. Adjusted Spearman’s correlations were computed with ppcor (v1.1) under R environment. Mann–Whitney U test was used for comparing two groups. Age and sex were used as covariates in all the analyses of the cohort data. Adjusted Spearman’s correlations were computed with ppcor (v1.1) under R environment (v4.2.1). Gene Set Enrichment Analysis was performed using Metascape [39] on significant genes negatively associated with hippurate in both tissues (liver or plasma).

#### 2.6.2 Cellular data

Cell biology and metabolomics quantification: If not stated otherwise, all data were presented as mean ± SEM calculated from a minimum of four independent (*N* > 4) experiments. The group by group comparisons were performed by unpaired Mann–Witney test. Comparisons of more than two groups were performed by Krustal-Wallis with interaction and Bonferroni post hoc test. P values with *p* < 0.05 were considered statistically significant. Figures and statistical analyses were performed using GraphPad Prism (version 5.0.0 for Windows, GraphPad Software, Boston, Massachusetts USA). The R function “cor.test” using the Spearman’s method was used to test the association between paired samples, returning the correlation coefficient and its p-value. Figures were generated by ggplot2 and Heatmap functions. To model between-group variance and thus identify variables underpinning the group discrimination, we performed orthogonal partial least squares discriminant analysis (O-PLS-DA) with the R package “ropls” [40]. The minimal number of significant components for the models was determined using a permutation test with 1,000 iterations [41].

## 3 Results

### 3.1 Hippurate is inversely correlated to liver steatosis and hepatic inflammation response in patients living with obesity

Previous studies showed that circulating and urinary hippurate negatively associate with MASLD [23,25], and we tested whether intracellular hippurate associates (negatively or not) with NAS component phenotypes derived from gold standard histopathological scoring and metabolic phenotypes in humans. This was analysed through profiling hippurate by ultra-performance liquid chromatography coupled to high-resolution mass spectrometry (UHPLC-HRMS) in liver and serum of 318 patients recruited to the Biological Atlas of Obesity (ABOS) study, as part of PreciNASH [31].

**Figure 1:**
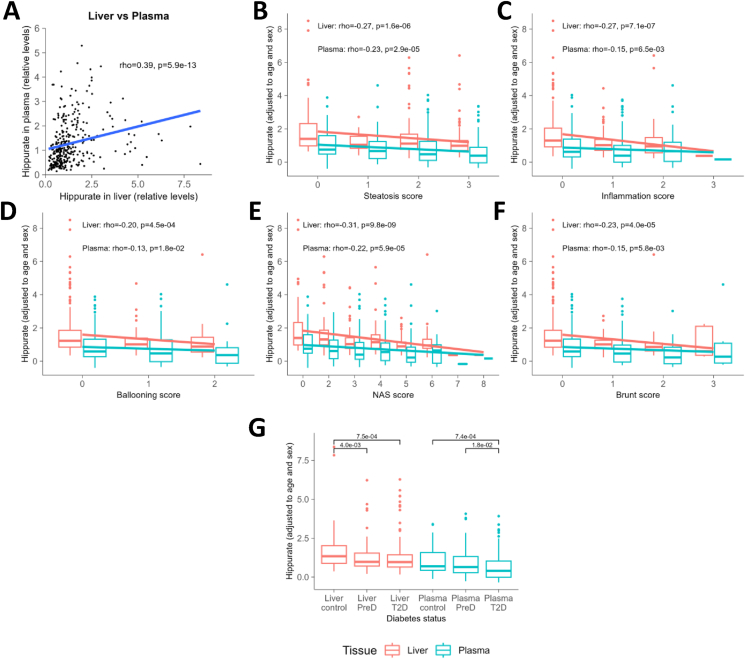
**Hippurate is negatively associated with steatosis and inflammation biomarkers in 318 patients from the ABOS study (PreciNASH project).** (A) Association between hippurate detected in liver and plasma. (B–F) Spearman correlations between plasma or liver hippurate and MASLD phenotypes. (G) Comparison of plasma and liver hippurate levels across diabetic status. (H) Heatmap of MASLD variables associated with hepatic hippurate in the liver or the plasma, stratified by Hb1Ac (high Hb1Ac ≥ 6.5%). (B–H) Hippurate levels in the liver and the plasma were corrected by age and sex. *N* = 318 patients.

First, we confirmed that hepatic and plasma hippurate are positively associated (Figure 1A, partial Spearman correlation adjusted for age and sex, rho = 0.39, *p* = 5.9 × 10^−13^). We then tested the association between MASLD phenotypes and circulating or intra-hepatic hippurate, adjusting for age and sex through partial Spearman correlations, and showed that, as expected from their correlation, both hepatic and plasma hippurate are significantly negatively associated with steatosis, inflammation, ballooning NAS and brunt scores (Fig. S1 and Figure 1B–F). Interestingly, the association with intra-hepatic hippurate was more significant for each of the tested phenotypes, with inflammation being more significant than steatosis. Furthermore, the levels of hippurate are reduced in patients with type 2 diabetes compared to patients with normoglycemia or impaired glucose tolerance, independently of age and sex (Figure 1G). Altogether our results confirm that hippurate is a microbiome-derived marker of metabolic health and show that intra-hepatic hippurate has stronger effect sizes than circulating hippurate further suggesting that intra-hepatic hippurate may have a direct protective role against MASLD phenotypes.

### 3.2 Hippurate is negatively correlated to the expression of genes involved in MASLD and MASH

**Figure 2:**
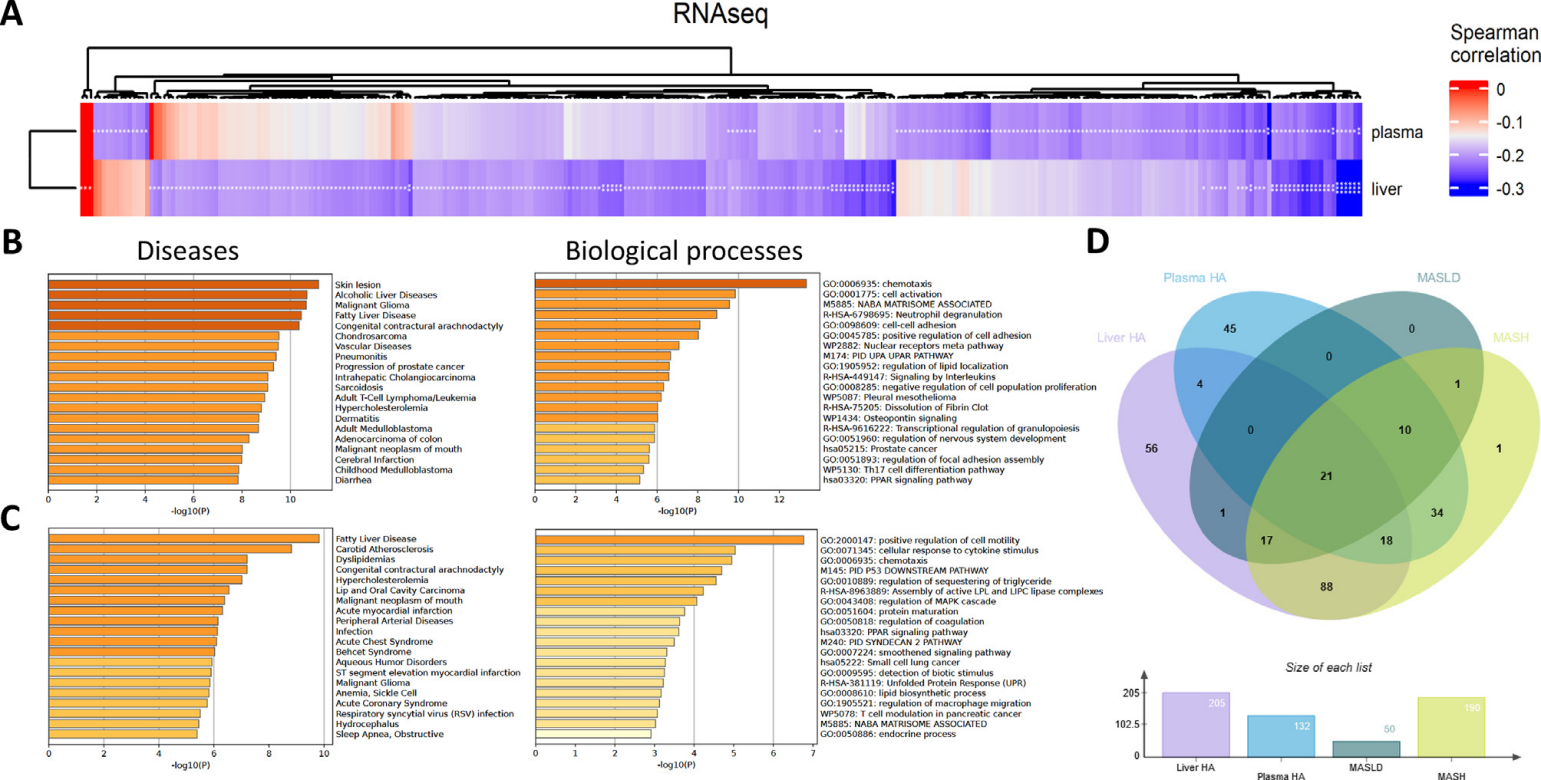
**Hippurate is negatively associated with transcripts of genes involved in non-MASH MASLD and MASH on 318 patients from ABOS cohort (PreciNASH project).** (A) Heatmap with significant associations between RNA transcripts and hippurate in both plasma and liver. Hippurate levels in the liver and plasma were corrected by age and sex. (B) Gene Set Enrichment Analysis (GSEA) performed using Metascape on significant genes negatively associated with hippurate in the liver. (C) GSEA performed using Metascape on significant genes negatively associated with hippurate in the plasma using DisGeNET database of gene–disease associations. (D) Venn diagram showing RNA transcripts negatively correlated to hippurate levels in the liver (purple), in the plasma (Blue), and RNA transcripts significantly related to non-MASH MASLD (Green) and MASH (yellow). (For interpretation of the references to colour in this figure legend, the reader is referred to the Web version of this article.)

To further investigate the protective role of hippurate in MASLD, we analysed global gene expression in liver via bulk RNA sequencing (RNA-seq) generated for these participants. The transcriptomics analysis revealed 208 and 132 genes whose expression is significantly associated with hippurate levels in the liver and in the plasma, respectively (Figure 2A), 43 of them being in common (Table S2). We further tested whether any of the 205 genes negatively associated with liver hippurate and 132 with plasma hippurate which were correlated with liver disease (Figure 2D [42]). We found that 49 (39 for liver hippurate and 31 for plasma hippurate) are associated with non-MASH MASLD and 188 (144 for liver hippurate and 83 for plasma hippurate) with MASH (Table S3).

We then performed a gene set enrichment analysis (GSEA, Figure 2B–C [43]), which showed that the genes that negatively correlated with hippurate in the liver (Figure 2B) are involved in skin lesions (UMLS: C0037284, Log10(p) = −11.00), alcoholic liver diseases (UMLS: C0023896, Log10(p) = −11.00), fatty liver diseases (UMLS: C4529962, Log10(p) = −10.00), vascular diseases (UMLS: C0042373, Log10(p) = −9.30), intrahepatic cholangiocarcinoma (UMLS: C0345905, Log10(p) = −8.90) and hypercholesterolemia (UMLS: C0020443, Log10(p) = −8.60). These genes are involved in the biological processes of chemotaxis (GO:0006935, Log10(p) = −13.20), cell activation (GO:0001775, Log10(p) = −9.70), regulation of lipid localization (GO:1905952, Log10(p) = −6.54) and signalling of interleukins (GO:R-HSA-449147, Log10(p) = −6.49) (Table S4).

Hippurate in plasma (Figure 2C) is negatively correlated with fatty liver disease (UMLS: C4529962, Log10(q) = −5.20), atherosclerosis (UMLS: C0577631, Log10(q) = −4.60), dyslipidaemias (UMLS: C0242339, Log10(q) = −3.20), hypercholesterolemia (UMLS: C0020443, Log10(q) = −3.20) and peripheral arterial diseases (UMLS: C1704436, Log10(q) = −2.70). These genes are involved in the processes of cell mobility (GO:2000147, Log10(P) = −6.72), cell response to cytokine (GO:0071345, Log10(P) = −5.00), chemotaxis (GO:0006935, Log10(P) = −4.92), regulation of sequestering triglyceride (GO:0010889, Log10(P) = −4.54), assembly of active LPL and LIPC (Reactome: R-HSA-8963889, Log10(P) = −4.22), regulation of MAPK pathway (GO:0043408, Log10(P) = −4.03), PPAR signalling pathway (KEGG: hsa03320, Log10(P) = −3.60) and lipid biosynthesis (GO:0008610, Log10(P) = −3.14) (Table S5).

Altogether, our association results in a cross-sectional study with severe obesity with a spectrum of MASLD phenotypes suggests a direct contribution of hippurate on MASLD pathophysiology. We next sought to validate this experimentally in hepatocytes.

### 3.3 Hippurate uptake in cultured hepatocytes impacts their metabolome and inhibits triglyceride storage

**Figure 3:**
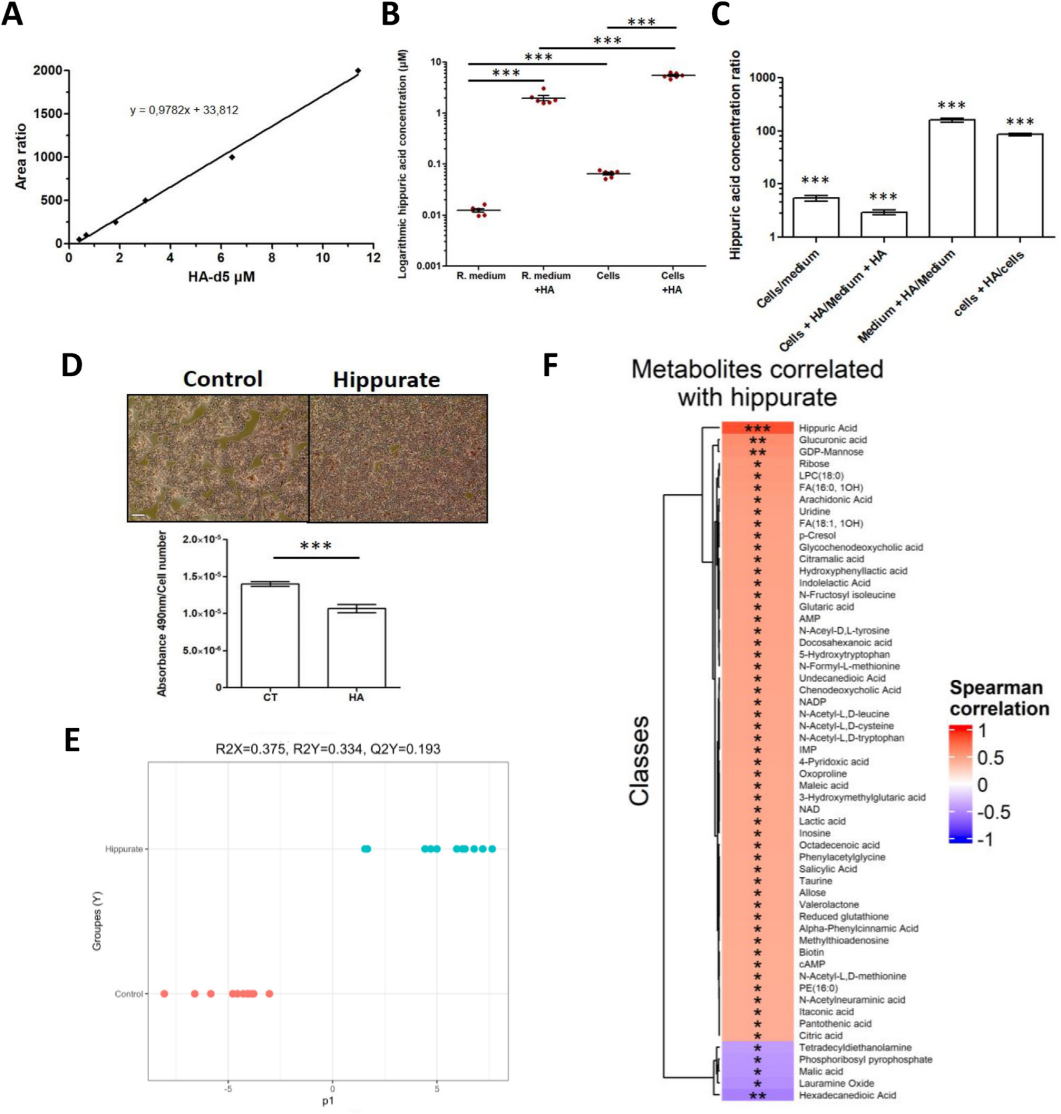
**Hippurate is uptaken in hepatocytes (IHH), inhibits their triglyceride storage and impacts their metabolome** (A) Calibration curve for isotopic quantification of hippurate using a hippurate-d5 isotopic standard, used to quantify native hippurate in samples. (B) Logarithmic quantification of hippurate present in residual medium (*R. medium*), residual medium supplemented with 500 μM hippurate (*R. medium* + HA), cell lysate (Cells), and lysate of cells treated 1 h with 500 μM hippurate (Cells + HA). (C) Ratio of hippurate concentrations between the different conditions in (B). (D) Representative pictures of immortalised human hepatocytes (IHH) cells after staining and quantification with 490 nm absorbance representing the triglyceride quantity inside cells. Scale bar represents 500 μm. (E) O-PLS-DA score plot vs class membership: Control (CT) and hippurate-treated IHH (HA) (1 predictive component and 0 orthogonal component, R^2^_Y_ = 0.334, Q^2^_Y_ = 0.193, pQ^2^_Y_ = 0.033). (F) Spearman correlation heatmap showing metabolites that significantly correlate to treated (HA) or untreated cells (CT). *N* = 4–6 independent replicates. *N* = 4–6 independent replicates.

Using a targeted metabolomics method, we measured intracellular hippurate and hippurate in the residual medium and compared it with our calibration curve (Figure 3A). Basal hippurate levels are very low in the residual medium and cells in the absence of additional exogenous hippurate, respectively 0.01 μM and 0.06 μM (Figure 3B), only reflecting the necessary presence of hippurate and benzoate in the medium [12551 - Williams' Medium E], and subsequent conjugation of benzoate with glycine to produce hippurate in hepatocytes [28]. Hepatocytes in both control and hippurate-treated (500 μM) conditions have a higher hippurate concentration than the residual medium (respectively 0.05 μM in control conditions and 5.4 μM in hippurate-treated conditions), suggesting its transport and accumulation from the medium to the cells (Figure 3C).

To confirm hippurate may causally impact MAFLD as suggested by our clinical study, we treated immortalised human hepatocytes (IHH) with hippurate for 24 h and assessed intracellular triglyceride accumulation using Oil Red-O. Hepatocytes treated with hippurate (HA) showed 24% lower levels of triglycerides than control cells (CT) (Figure 3D, *p* = 8.99 × 10^−06^). This is consistent with the literature [25] and supports the associations reported in Figure 1, bringing to light a negative correlation between hippurate concentration and hepatic steatosis.

To understand how hippurate downregulates lipid accumulation in hepatocytes, we then performed untargeted metabolomics analysis using UHPLC-HRMS on IHH cellular extracts, reporting the annotation of 250 metabolites and lipids (Table S6). We then built an orthogonal partial least squares discriminant analysis (O-PLS-DA) model, which efficiently classified samples based on hippurate treatment groups and visualised an impact of hippurate (500 μM) on the metabolome of treated cells (Figure 3E, R^2^_Y_ = 0.334, Q^2^_Y_ = 0.193, pQ^2^_Y_ = 0.033). The hippurate treatment is significantly correlated with specific metabolites (Figure 3F, Table S7) and displays negative correlations with phosphoribosyl pyrophosphate involved in the biosynthesis of purines and pyrimidines (AMP, GMP, IMP, NAD+, NADP+) [44]. Hippurate is positively correlated with second messengers such as cyclic adenosine monophosphate (cAMP) with protective effects against MAFLD and ALD [45], methylthioadenosine and IMP which inhibit the production of TNF-alpha and enhance interleukin-10 production [46,47]. Glutathione (GSH) is a powerful antioxidant limiting lipid peroxidation [48,49] is shown to be reduced with the hippurate treatment. NAD+/NADP+, involved in a wide range of metabolic reactions and whose deficiency increases oxidative stress [50], indolelactic acid (ILA) derived from tryptophan, which can inhibits pro-inflammatory cytokine IL-8 via the transcription factor AHR [51,52], and salicylic acid which inhibits COX1 and Cox-2 synthesising pro-inflammatory prostaglandins [53,54] are all negatively associated to hippurate.

Hippurate supplementation showed to upregulate the production of metabolites involved in energy homeostasis, such as citrate [55], intracellular glucose and cAMP which is a secondary messenger involved in several biochemical processes (such as sugar and lipid metabolisms) [45]. Hippurate is also positively correlated to biotin (vitamin B8), important in lipid and carbohydrate anabolism and normal protein synthesis [56,57], taurine, which is a mediator of cell homeostasis [58,59] and plays also an important role in energy metabolism, especially in metabolic syndrome context [60,61], and also NAD/NADP [50]. 3-hydroxymethylglutarate, an antilipidemic agent lowering cholesterol and triglycerides [62] and Inosine-Monophosphate (IMP), the precursor of two energy molecules, GMP and AMP [63] are linked to hippurate.

Our phenotypic and metabolomic results suggest that hippurate could be a beneficial regulator of energy homeostasis and lipid metabolism in hepatocytes.

### 3.4 Hippurate rescues insulin resistance induced by 24-hour chronic insulin exposure

**Figure 4:**
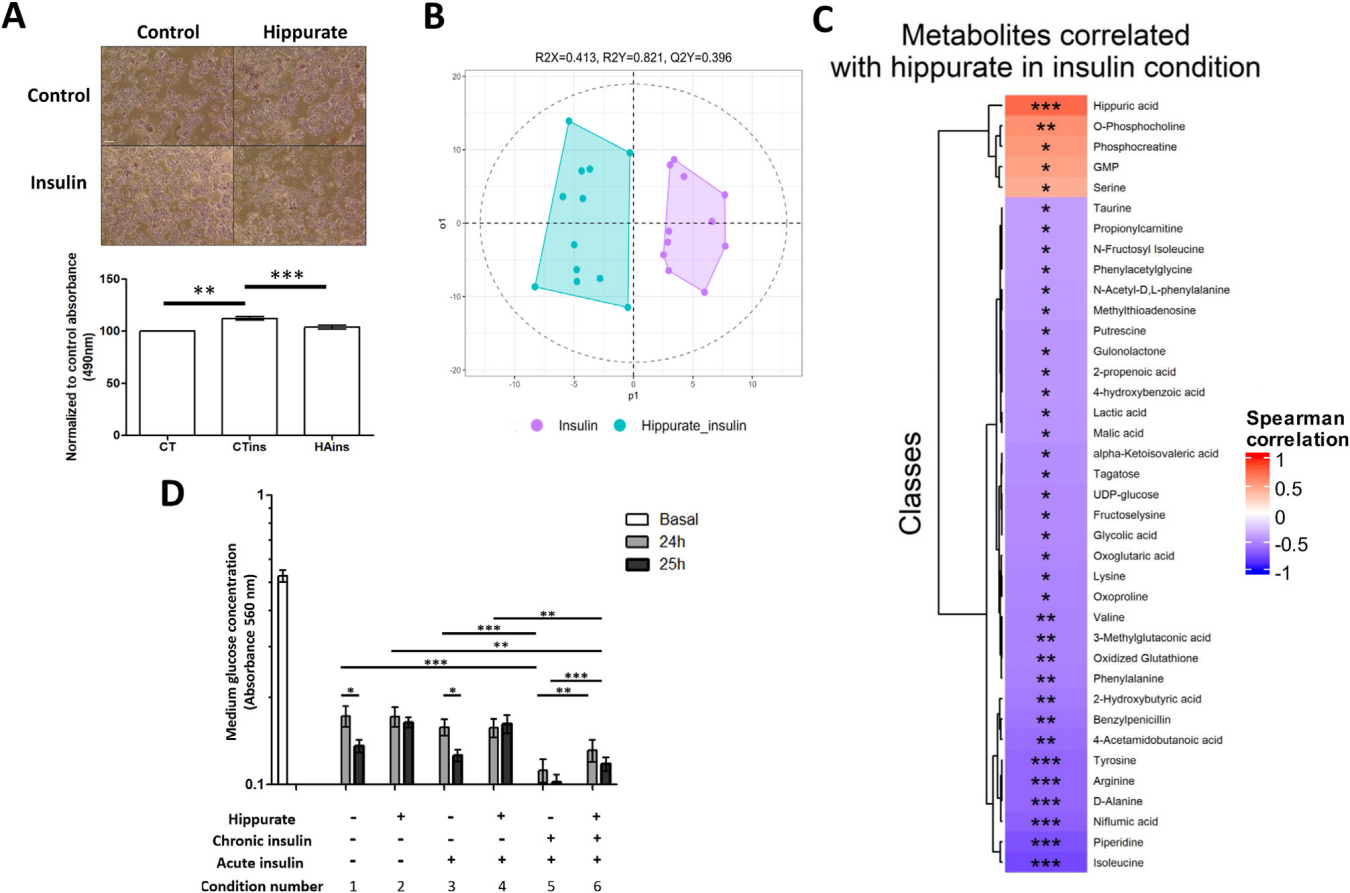
**Hippurate rescues triglyceride storage, glucose uptake and hepatocyte metabolome in the context of insulin resistance (treated 24 h with 100 nM insulin)** (A) Representative picture of immortalised human hepatocytes (IHH) cells after staining and quantification with 490 nm absorbance representing the triglyceride quantity inside cells. Scale bar represents 500 μm. (B) O-PLS-DA showing the repartition of the different samples: Insulin treated cells (CTins) and hippurate-insulin-treated cells (HAins) (1 predictive component and 1 orthogonal component, R^2^_Y_ = 0.821, Q^2^_Y_ = 0.396, pQ^2^_Y_ = 0.011). (C) Spearman correlation heatmap showing metabolites that significantly correlate to (supplementary data 2C) the insulin-treated cells (CTins) and hippurate-insulin-treated cells (HAins) from 250 metabolites (D) Insulin-stimulated glucose uptake by IHH cell quantified by measuring glucose left in the medium at *t* = 0 (Basal, the same for all conditions), *t* = 24 h and *t* = 25 h. *N* = 4–6 independent replicates.

To test the effect of hippurate on insulin-resistant hepatocytes, we treated IHH cells with insulin (100 nM) for 24 h with and without hippurate (500 μM) before quantifying lipid accumulation (Figure 4A), insulin-stimulated glucose uptake (Figure 4D) and its impact on the metabolome (Figure 4B–C). Hippurate reduces lipid accumulation by 22% (*p* = 1.35 × 10^−10^) in the basal condition compared to control (CT), which is similar to Figure 3D, and by 8% (*p* = 2 × 10^−4^) in the insulin condition compared to control (CTins) (Figure 4A). This significantly reduced triglyceride storage caused by hippurate supplementation is not a reflection on its abundance as the rescued triglyceride concentration with the addition of hippurate is not statistically significant when compared to its control (*p* = 0.26). This result supports the idea that hippurate is a beneficial regulator of lipid storage in the context of insulin resistance [30].

By using untargeted metabolomics with UHPLC-HRMS to profile the metabolome of IHH depending on hippurate treatment (500 μM) in insulin resistance conditions (24 h of 100 nM insulin treatment), we were able to discriminate between treatment conditions (Figure 4B, R^2^_Y_ = 0.821, Q^2^_Y_ = 0.396, pQ^2^_Y_ = 0.011). The discrimination between these two conditions is stronger than the discrimination in control condition (Figure 3E) which suggests that the impact of hippurate could be more important under insulin conditions.

Metabolites associated with hippurate treatment under 24 h chronic insulin exposure were then visualised through a Spearman correlations heatmap (Figure 4C, Table S7). The analysis shows a high number of significant negative correlations with hippurate, suggesting an inhibitory effect of hippurate on multiple pathways.

Hippurate normalises most of the metabolite profiles that are up-regulated by chronic insulin, acting like a brake on insulin effect (Fig. S2). This finding is also consistent with our observation that hippurate (500 μM) reduces glucose uptake (Figure 4D). Hippurate (condition 2, 4 and 6) inhibits the cellular glucose uptake with or without 1 h of 200 nM insulin. This effect is not observed when hippurate is not present in the media, as glucose uptake (insulin-dependent and independent; conditions 1 and 3) was significantly increased between 24 h and 25 h. Cells with/without hippurate treated chronically with insulin (conditions 5 and 6) did not display statistically significant acute insulin-dependent glucose uptake. Although this is the case, glucose uptake was as expected, significantly increased compared to acute treatment with insulin. Hippurate reduces glucose internalisation, even during the 24 h of exposure to insulin (condition 6). Altogether, hippurate leads to a relative normalisation of chronic insulin-stimulated glucose uptake, with a similar pattern to lipid accumulation and cellular metabolome profile.

## 4 Discussion

With MASLD and liver disease prevalence growing with the obesity epidemic, it is becoming crucial to understand the mechanisms underpinning whether an obese individual will develop MASLD, or not. We have previously shown that the microbiome plays an important role in MASLD [17], which suggests that beneficial microbiome-derived metabolites and/or postbiotics could provide diagnostic and therapeutic avenues for MASLD [20,21]. In particular, we and others have shown that hippurate improves metabolic health in mouse models [23,25,30]: in humans, circulating hippurate has been positively associated with microbiome richness and with metabolic health, especially in patients living with obesity [23,29,30]. Based on these reports, hippurate can be proposed as a biomarker of human metabolic health.

In this study, we show for the first time that hepatic hippurate not only correlates with plasma hippurate but also negatively correlates with key hallmarks in MASLD such as inflammation, hepatic steatosis and ballooning, but also with type 2 diabetes with stronger effect sizes than circulating hippurate. Consistently, hippurate is inversely correlated with the expression in liver of 49 genes involved in the development of non-MASH MAFLD and 188 in MASH. Among these genes, several are involved in the pathophysiology of steatosis and consequences. For example, PPARD codes for the peroxisome proliferator-activated receptor delta, a ligand-dependant transcription factor whose regulation activates genes involved in adipogenesis and inflammation [64] such as CIDEC (cell death-inducing DFFA-like effector c), a protein which promotes intracellular triglyceride storage [65]. LPL (lipoprotein lipase) expression is almost absent in normal adult livers but is upregulated by obesogenic molecules such as free fatty acid, leptin, and interleukin-6 [66] and is negatively correlated to hippurate. TNFRSF12A (Tumor necrosis factor receptor superfamily member 12 A) is also nearly undetectable in normal adult livers but substantially increased in hepatic progenitor cells during chronic liver diseases, including MASH, alcoholic liver disease, and chronic hepatitis C [67]. Finally, FABP4 and FAPB5 (Fatty acid-binding protein 4 and 5) are associated with inflammation, liver fibrosis and insulin resistance [68,69]. These genes and the 201 others revealed that liver and plasma hippurate levels are inversely correlated with MASLD, lipid deregulation and diseases due to chronic inflammation such as sarcomas, gliomas and carcinomas [70].

These results suggest that some of the hippurate whole-body effects on MASLD observed in humans and mice may well be at least partly explained by a direct positive role on hepatocyte function. Improving or protecting liver function is one of the key strategies to maintain the homeostasis of individual, as the liver is a crucial metabolic and detoxifying organ [17,71,72]. Our results on IHH show that the hippurate is not just negatively associates with MASLD and MASH but may also be an active operator of hepatic metabolic homeostasis. In hippurate-treated hepatocytes, triglyceride storage and glucose uptake are reduced. This effect is present in physiological conditions, but also in conditions of insulin resistance, such as those found in patients with obesity and/or with MAFLD [73]. When administered chronically, hippurate subtly modifies the metabolome of hepatocytes by increasing the relative abundance of metabolites involved in energy homeostasis and decreasing those involved in inflammation and insulin resistance. For instance, starting with amino acids, we observe negative correlations with BCAAs, such as isoleucine, whose concentrations are commonly increased in the blood of diabetic mice, rats, and humans, and a one-day deprivation improves insulin sensitivity in mice [[74], [75], [76]]. Valine, another BCAA, impairs glucose tolerance in the context of a high-fat diet in mice, in particular through its conversion into 3-hydroxyisobutyrate (3-HIB) [74,77] and is negatively associated to hippurate. Likewise, aromatic amino acid phenylalanine associated with insulin resistance by modification of the insulin receptor beta (IRβ) [75,78], and tyrosine which plays a central role for the function of many proteins but may be positively correlated to insulin resistance and is elevated in liver cirrhosis [79,80] are also negatively correlated to hippurate. Hippurate also anticorrelates with metabolites linked to inflammation such as UDG, which can also act as a pro-inflammatory signalling molecule under stressful conditions by agonism of P2Y14 receptor [81] and oxidized glutathione for which the ratio of reduced glutathione to oxidized glutathione is an indicator of cellular health [82]. In insulin stimulation, hippurate is also negatively correlated to toxic metabolites. For example, alpha-ketoisovaleric acid, a metabolic biomarker for the development of diabetes [75,83] and phenylacetylglycine, a biomarker of phospholipidosis [84]. Hippurate is also positively associated with metabolites involved in preserving available ATP, such as phosphocreatine [[85], [86], [87]], or protecting hepatocytes from oxidative stress induced by a high-fat diet, such as serine [[88], [89], [90]].

Our study provides valuable insights into the potential effects of hippurate, however, several limitations should be noted. One of the limitations is the absence of gut microbiome data, which restricts our ability to document the potential protective effects of hepatic and plasma hippurate with microbial species, genes or functions, or the presence of dysbiosis as previously performed by us in Brial et al., 2021. Further studies integrating microbiome analyses would be beneficial to explore possible correlations between intra-hepatic hippurate and specific microbial profiles, shedding light on its role in overall metabolic and intestinal health. Another limitation relates to the observational nature of the human study, which validates previous observations about the negative association between circulating Hippurate and MASLD/T2D phenotypes and establishes novel associations. However, this observational setting does not establish causality between hippurate and MASLD/T2D. Beyond the treatments performed in cellular systems, further research is needed, ideally through mechanistic studies, to better understand the mechanism and therapeutic potential. A third limitation is the relative, rather than absolute, dosage measurement of hippurate. Hippurate levels in this study are compared between groups (non-MASLD, MASLD non-MASH, MASH) without precise measures of absolute concentrations. However, hippurate concentration varies significantly based on factors such as age, sex, body composition, and diet, making it challenging to determine an effective pharmacological dose of hippurate. This variability also limits the accuracy of therapeutic assessments in future models. Finally, the use of immortalized cells, although advantageous for proof-of-concept studies and certain aspects of this thesis project, also poses specific challenges. These cells undergo genetic and phenotypic modifications, which can result in a loss of functional specificity. For instance, progressive degeneration, measured by passage number (cell division count), may impair the production of key compounds like insulin and affect cellular response to stress or treatments. To validate these results, it would be beneficial to replicate findings using models that better reflect human physiology, particularly by using primary cells and organs-on-chip systems.

Altogether, our clinical results suggest that hepatic hippurate is a more precise marker of lower MASLD phenotype values than circulating hippurate, which is currently reported, being more routinely accessible. This result is consistent with the idea that intra-hepatic hippurate plays a protective role against MASLD in the targeted organ, which is also supported by the number of non-MASH MASLD and MASH genes anticorrelated with hepatic hippurate compared to plasma hippurate. This protective role is then further confirmed by our preclinical experiments in IHH showing that hippurate confers protection against MASLD: we show that not only hippurate directly inhibits triglyceride storage in both baseline and chronic 24-hr insulin conditions and rescue the effect of chronic insulin, but it also improves insulin-stimulated glucose update in condition of insulin resistance triggered by 24-hr chronic insulin pretreatment. Our study suggests that even under physiological conditions, hippurate is beneficial for energy (lipid and sugar metabolism) and inflammatory homeostasis which inhibits liver steatosis, while in chronic insulinemia conditions, that characterizes obesity and pre-diabetes, it would act as an antagonist to the effects of insulin resistance.

In conclusion, we bring novel clinical and preclinical evidence further characterising hippurate as a beneficial host-microbiome co-metabolite for MASLD. Our work further reinforces the view that hippurate could therefore be a prime postbiotic candidate for clinical studies, particularly for the prevention of MASLD in patients with obesity. This opens perspectives for more in-depth exploration of the molecular mechanism behind the postbiotic effects of hippurate in preclinical and clinical settings.

## CRediT authorship contribution statement

**Maxime Deslande:** Writing – original draft, Methodology. **Francesc Puig-Castellvi:** Formal analysis, Conceptualization. **Inés Castro-Dionicio:** Methodology, Investigation. **Romina Pacheco-Tapia:** Methodology, Investigation. **Violeta Raverdy:** Methodology, Investigation. **Robert Caiazzo:** Methodology, Investigation. **Guillaume Lassailly:** Methodology, Investigation. **Audrey Leloire:** Methodology. **Petros Andrikopoulos:** Methodology, Investigation. **Yasmina Yahoul:** Methodology, Investigation. **Nawel Zaïbi:** Methodology, Investigation. **Bénédicte Toussaint:** Methodology, Investigation. **Frédérik Oger:** Methodology. **Nicolas Gambardella:** Methodology, Investigation. **Philippe Lefebvre:** Methodology, Investigation. **Mehdi Derhourhi:** Methodology, Investigation. **Souhila Amanzougarene:** Methodology, Investigation. **Bart Staels:** Supervision, Methodology, Investigation, Funding acquisition. **François Pattou:** Writing – review & editing, Supervision, Methodology, Investigation, Funding acquisition, Conceptualization. **Philippe Froguel:** Writing – review & editing, Supervision, Methodology, Investigation, Funding acquisition, Conceptualization. **Amélie Bonnefond:** Writing – review & editing, Supervision, Methodology, Investigation, Funding acquisition, Conceptualization. **Marc-Emmanuel Dumas:** Writing – review & editing, Writing – original draft, Supervision, Project administration, Methodology, Investigation, Funding acquisition, Conceptualization.

## Declaration of competing interest

The authors declare that they have no known competing financial interests or personal relationships that could have appeared to influence the work reported in this paper.

## Data Availability

Data will be made available on request.

## Glossary

**Host-microbial co-metabolite**: A compound whose production relies on enzymatic reactions occurring in both the microbiota and the host. For example, Hippurate is produced by the host’s hepatocytes from the gut microbial metabolite benzoate
**GSEA analysis**: A method used to determine whether the number of significant genes are statistically over-represented in predefined sets, where its set is associated with a particular biological process, disease, or cellular compartment
**Metabolic health**: The metabolic state characterised by absence of metabolic syndrome, severe obesity and/or type 2 diabetes, with key metabolic markers in a healthy range such as fasting plasma glucose, blood pressure, high-density lipoprotein cholesterol and circulating triglycerides [91]
**Orthogonal Projections to Latent Structures Discriminant Analysis (O-PLS-DA)**: A multivariate dimensionality-reduction method for separating different study groups
**Postbiotic**: A substance, metabolite, gene product or cellular component, originally derived from the microbiota with potentially beneficial properties for the host. Postbiotics serve as bioactive compounds as an alternative to prebiotics or probiotics
**Transcriptomics**: The study of the RNA (*transcript*) levels in a given cell, tissue, organ, organism or community under specific conditions. Differences in the RNA levels give information, among others, about how the system study is regulated at the gene level and how it responds to stress
**Ultra-High-Performance Liquid Chromatography/High-Resolution Mass Spectrometry (UHPLC/HRMS)**: A high-throughput method using targeted and untargeted metabolomics that allows the quantitative and qualitative characterisation, respectively, of small molecules in different matrices

## Abbreviations

3-HIB: 3-hydroxyisobutyrate
ABOS: Biological Atlas of Obesity
ALD: Adrenoleukodystrophy
AMP: Adenosine monophosphate
ASD: Authentic Analytical Standard
ATP: Adenosine triphosphate
BCAA: Branched amino acid
cAMP: Cyclic adenosine monophosphate
CIDEC: Cell death-inducing DFFA-like effector c
COX: Cyclooxygenase
DDA: Data dependent mode
DMSO: Dimethyl sulfoxide
ESI: Electrospray ionization
FABP: Fatty acid-binding protein
GMP: Guanosine Monophosphate
GO: Gene ontology
GSEA: Gene Set Enrichment Analysis
GSH: Reduced glutathione
HA: Hippurate
HA-D5: Hippurate-d5
HbA1c: Glycated haemoglobin
HCD: Higher-energy collisional dissociation
HEPES: 4-(2-hydroxyethyl)-1-piperazineethanesulfonic acid
HFD: High-fat diet
HRP: Horseradish Peroxidase Concentrate
IHH: Immortalised human hepatocytes
ILA: Indolelactic acid
IMP: Inosine Monophosphate
InChIKeys: International Chemical Identifier
IRβ: Insulin receptor beta
ISD: Internal standard
IUPAC: International Union of Pure and Applied Chemistry
LC/MS: Liquid chromatography coupled to mass spectrometry
LPL: Lipoprotein lipase
MASH: Metabolic dysfunction-associated steatohepatitis
MASLD: Metabolic dysfunction-associated steatotic liver disease
MeOH: Methanol
MS1/2: Tandem mass spectrometry
NAD/NADP: Nicotinamide adenine dinucleotide/Nicotinamide adenine dinucleotide phosphate
NAS: MAFLD activity score
O-PLS-DA: orthogonal partial least squares discriminant analysis
PBS: Phosphate-buffered saline
PPARD: Peroxisome proliferator-activated receptor delta
PQN: Probabilistic quotient
QC: Quality control
RNA-seq: RNA sequencing
RSD: Relative standard deviation
SEM: Standard Error of the Mean
TCA cycle: Tricarboxylic acid cycle
TNF: Tumor necrosis factor
TNFRSF12A: Tumor necrosis factor receptor superfamily member 12 A
UDG: Diphosphate glucose
UHPLC-HRMS: Ultra-high-performance liquid chromatography coupled to high-resolution mass spectrometry
UMLS: Unified Medical Language System

## Appendix ASupplementary data

Multimedia component 1 Table S1. PreciNASH cohort summary. Multimedia component 1

Multimedia component 2 Table S2. Hepatic genes correlated to HA. Multimedia component 2

Multimedia component 3 Table S3. Hepatic genes related to non-MASH-MAFLD and MASH. Multimedia component 3

Multimedia component 4 Table S4. Gene Set Enrichment analysis of genes correlated to liver levels of hippurate. Multimedia component 4

Multimedia component 5 Table S5. Gene Set Enrichment analysis of genes correlated to plasma levels of hippurate. Multimedia component 5

Multimedia component 6 Table S6. Metabolites identified by UHPLC-HRMS in IHH. Multimedia component 6

Multimedia component 7 Table S7. Hepatic metabolites significantly correlated with hippurate treatment in basal and chronic insulin conditions. Multimedia component 7
