# Supplementary material for: Intrahepatic levels of microbiome-derived hippurate associates with improved metabolic dysfunction-associated steatotic liver disease
Source: Mol Metab. 2024 Dec 31;92:102090. doi: 10.1016/j.molmet.2024.102090 (PMC11772989; doi:10.1016/j.molmet.2024.102090)
Supplement: Multimedia component 8 [file mmc8.pdf]

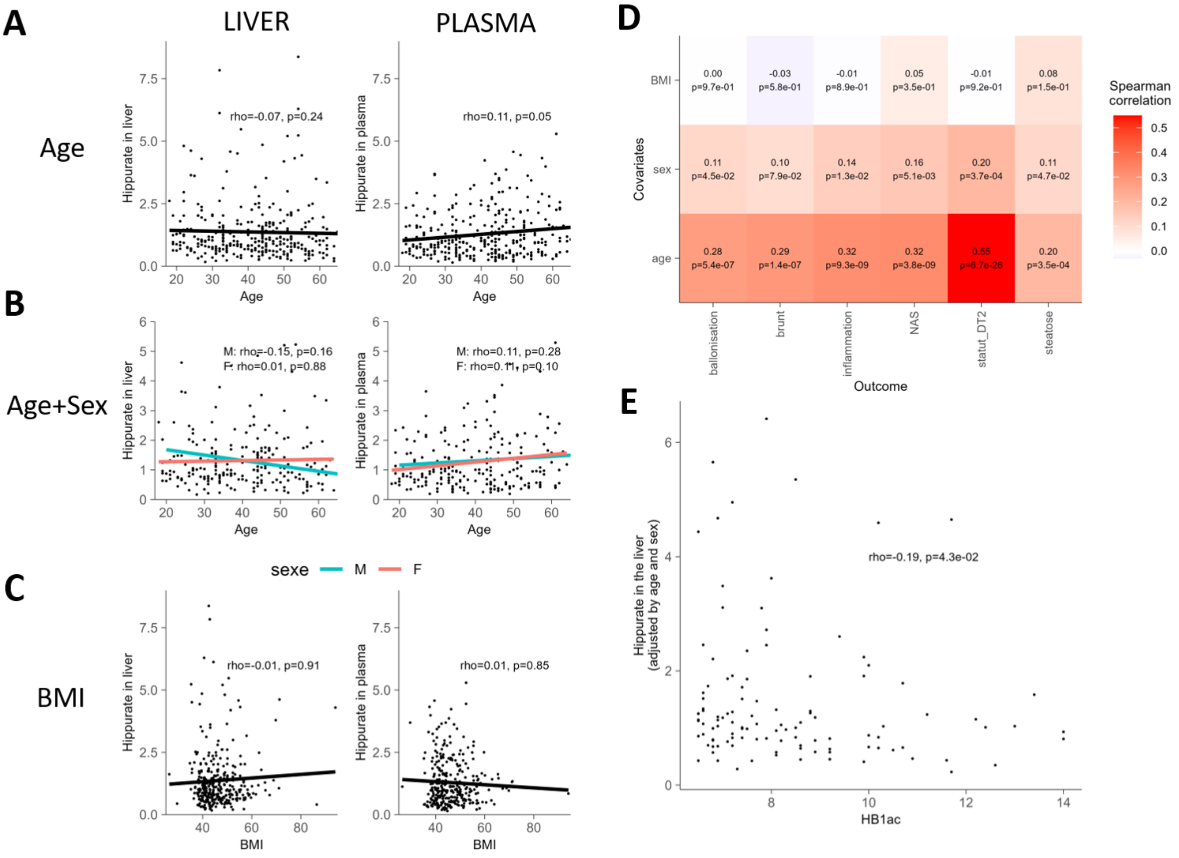

Figure S1 : Hippurate is correlated to age, sex and Hb1Ac but not BMI. Intra-hepatic hippurate (liver) and plasmatic hippurate (plasma) associations to (A) Age, (B) Age + Sex and (C) BMI (Body Mass Index). (D) Correlation heatmap between BMI, Age and Sex as covariates and MAFLD biomarkers. (E) Association between Hb1Ac and intra-hepatic hippurate N = 318 patients from ABOS cohort.

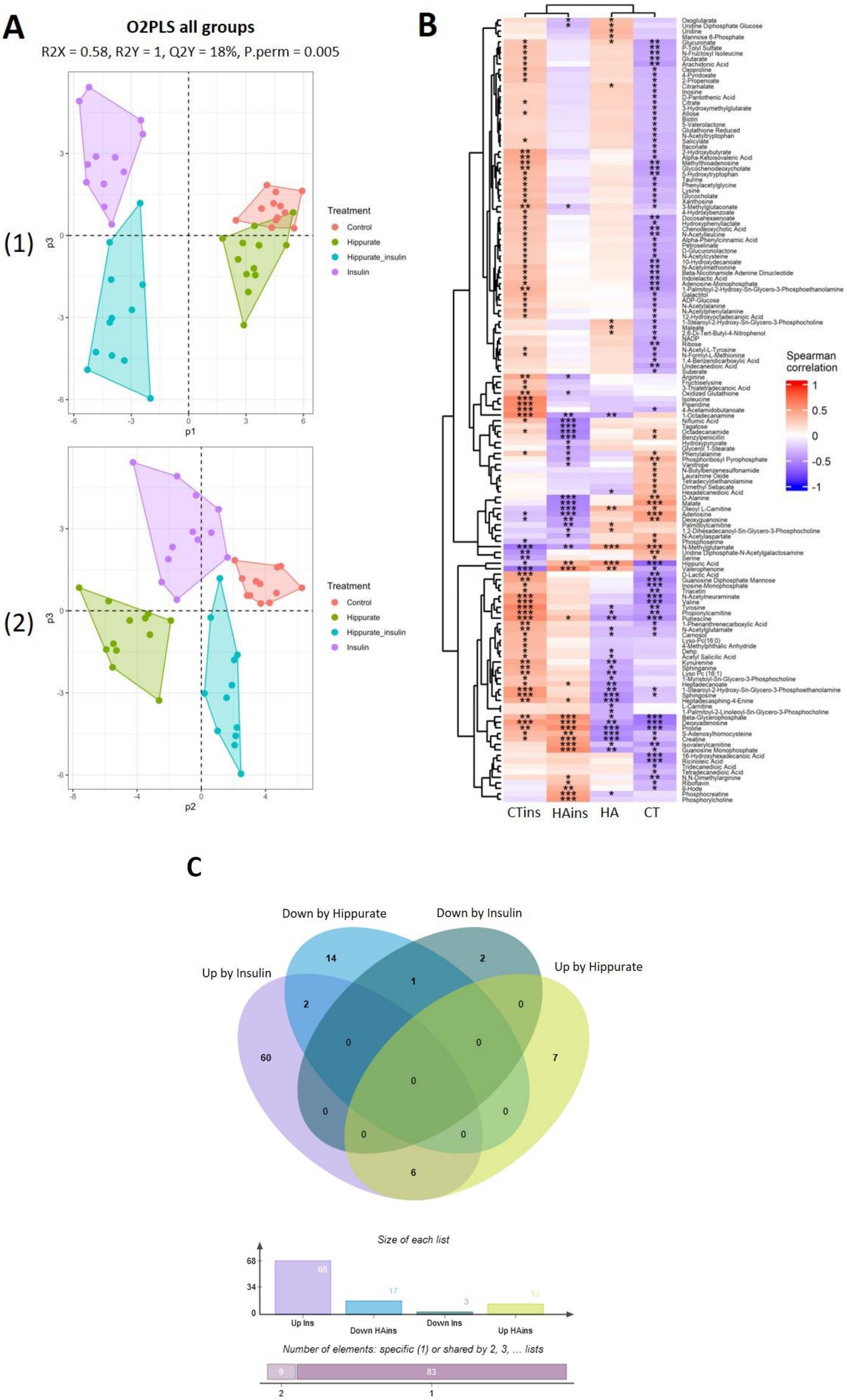
A
O2PLS all groups
R2X = 0.58, R2Y = 1, Q2Y = 18%, P.perm = 0.005
(1)
(2)
p1
p2
p3
Treatment
Control
Hippurate
Hippurate_insulin
Insulin
B
Spearman correlation
1
0.5
0
-0.5
-1
CTins
HAins
HA
CT
C
Down by Hippurate
Down by Insulin
Up by Insulin
Up by Hippurate
Size of each list
Up Ins
Down HAins
Down Ins
Up HAins
Number of elements: specific (1) or shared by 2, 3, ... lists

Figure S2 : **Hippurate impacts significantly IHH metabolome in normal and insulin resistance condition**. (A) O2PLS-DA analysis between 4 groups (Control, CT, hippurate treated cells, HA, insulin treated cells, CTins, and hippurate-insulin treated cells, HAins). (B) Spearman's correlation heatmap between these 4 groups. (C) Venndiagram representing metabolites positively correlated (up) or negatively correlated (down) by insulin (CTins) and hippurate (HAins) in insulin condition. The samples used here come from figures 3 and 4, which have been reprocessed to analyse the 4 groups at the same time.
